# Supplementary material for: Effects of Environmental Conditions on the Individual Architectures and Photosynthetic Performances of Three Species in Drosera
Source: Int J Mol Sci. 2023 Jun 6;24(12):9823. doi: 10.3390/ijms24129823 (PMC10298719; doi:10.3390/ijms24129823)
Supplement: Supplementary file 1 [file ijms-24-09823-s001.zip › ijms-2392073-supplementary.pdf]

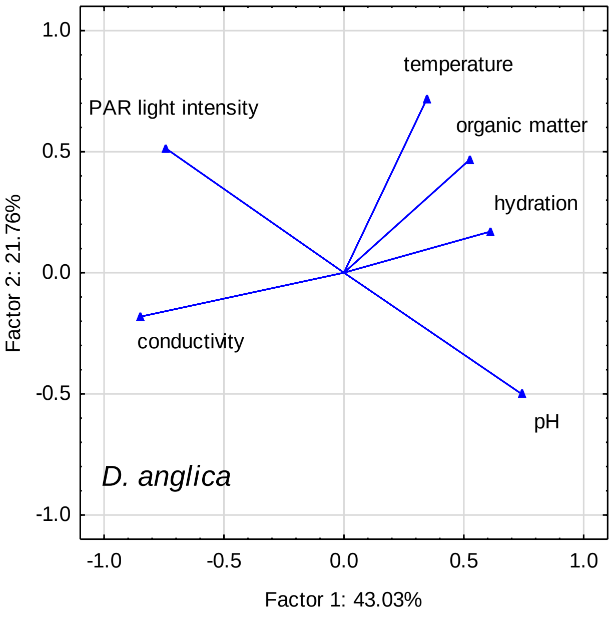

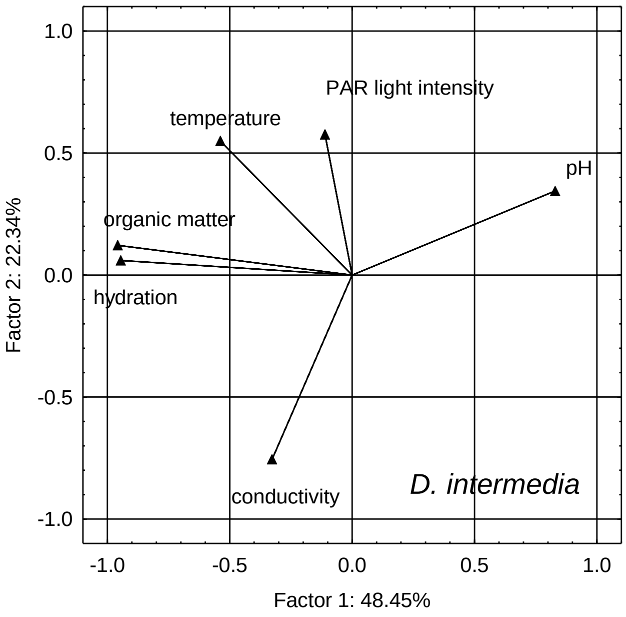

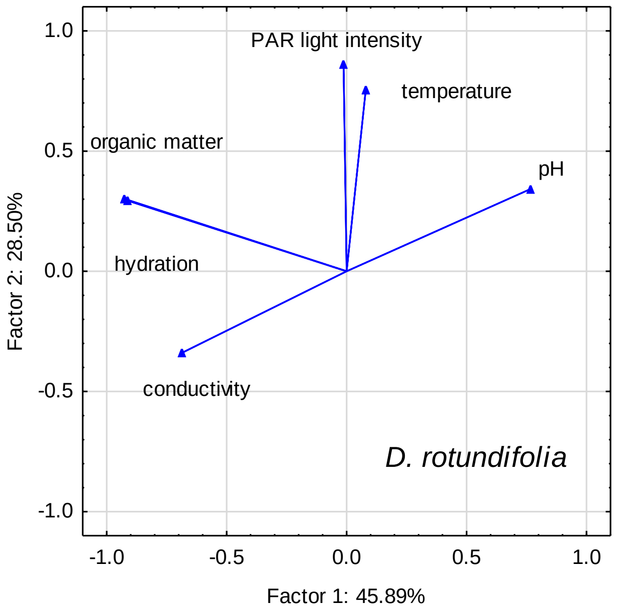

Factor loadings

Trait \ Factor	*D. anglica* 1	*D. anglica* 2	*D. intermedia* 1	*D. intermedia* 2	*D. rotundifolia* 1	*D. rotundifolia* 2
pH	0.740	-0.499	0.830	0.345	0.767	0.342
Conductivity	-0.848	-0.181	-0.327	-0.755	-0.688	-0.341
Temperature	0.346	0.717	-0.538	0.549	0.080	0.751
PAR light intensity	-0.741	0.513	-0.111	0.576	-0.013	0.858
Hydration	0.610	0.170	-0.945	0.060	-0.911	0.294
Organic matter	0.524	0.466	-0.957	0.122	-0.925	0.301

Figure S1. Factors differentiating habitat conditions of sundews according to factor analysis and factor loadings based on correlations of environmental traits with factors 1 and 2.

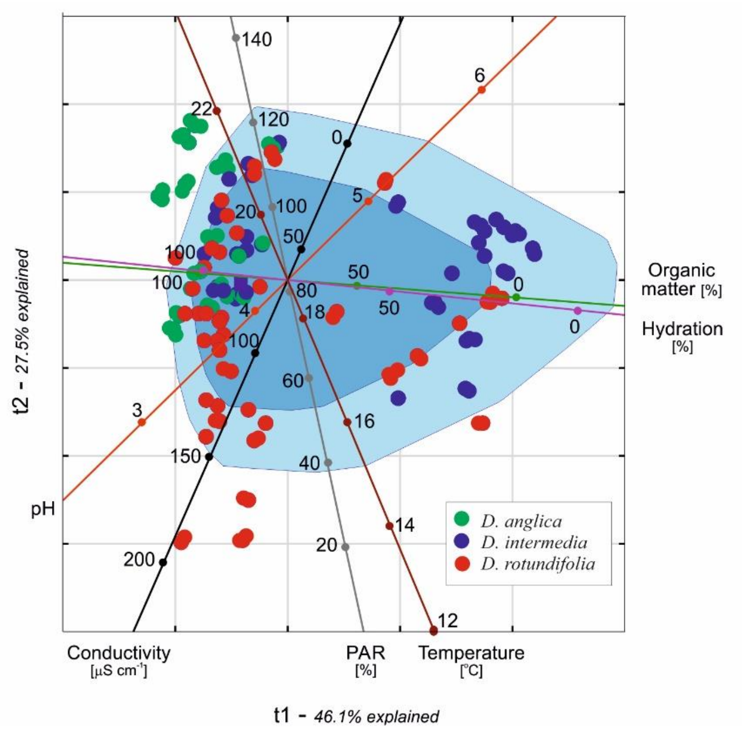

Figure S2. Variation in environmental conditions of *Drosera* according to Partial Least Squares (PLS) analysis.
